# Incorporating ENCODE information into association analysis of whole genome sequencing data

**DOI:** 10.1186/s12919-016-0040-y

**Published:** 2016-10-18

**Authors:** Taebeom Kim, Peng Wei

**Affiliations:** Department of Biostatistics, The University of Texas MD Anderson Cancer Center, Houston, TX 77030 USA

## Abstract

With the rapidly decreasing cost of the next-generation sequencing technology, a large number of whole genome sequences have been generated, enabling researchers to survey rare variants in the protein-coding and regulatory regions of the genome. However, it remains a daunting task to identify functional variants associated with complex diseases from whole genome sequencing (WGS) data because of the millions of candidate variants and yet moderate sample size. We propose to incorporate the Encyclopedia of DNA Elements (ENCODE) information in the association analysis of WGS data to boost the statistical power. We use the RegulomeDB and PolyPhen2 scores as external weights in existing rare variants association tests. We demonstrate the proposed framework using the WGS data and blood pressure phenotype from the San Antonio Family Studies provided by the Genetic Analysis Workshop 19. We identified a genome-wide significant locus in gene *SNUPN* on chromosome 15 that harbors a rare nonsynonymous variant, which was not detected by benchmark methods that did not incorporate biological information, including the T5 burden test and sequence kernel association test.

## Background

Genome-wide association studies (GWAS) have identified thousands of genetic loci robustly associated with a wide range of complex diseases and traits. However, there is a big gap between the disease heritability explained by GWAS-identified loci and that estimated from twin/family-based studies, leading to the so-called missing heritability [1]. To fill in this gap, recent genetic studies have shifted gear from GWAS investigating common single-nucleotide polymorphisms with a minor allele frequency (MAF) larger than 5 % to low frequency (MAF between 1 and 5 %) and rare variants (RVs with MAFs <1 %) afforded by the next-generation sequencing (NGS) technology. As a result of the relatively low cost of the whole exome sequencing (WES), the first wave of NGS-based association studies of complex diseases, for example, the Exome Sequencing Project (ESP), has primarily focused on the protein-coding regions of the human genome, that is, the exome, constituting approximately 1 % of the total genome. Although the WES has been extremely useful in identifying causal variants for Mendelian disorders, the success with WES-based association studies of complex diseases has been very limited thus far [2]. This is partly because of the limited statistical power afforded by the current sample size of WES studies, and partly because of the incomplete coverage of the human genome by the WES. To improve the power, many new statistical methods for analysis of RVs have been proposed in the past few years, including the T1/T5 burden tests (variant collapsing methods with a MAF threshold of 1 % or 5 %), sequence kernel association test (SKAT) [3], adaptive sum of powered score (aSPU) [4], among others; see Lee et al. [2] and Pan et al. [4] for recent reviews.

Thanks to the rapidly decreasing cost of whole genome sequencing (WGS), thousands of whole genome sequences have been generated [5], enabling researchers to go beyond the exome and survey RVs in the regulatory regions of the genome. However, with almost 100 times more variants and even smaller sample size in WGS than those in WES, it remains a significant challenge to analyze WGS data. To boost the power, we and others have previously proposed statistical methods to leverage external biological information, such as computational predictions of damaging effects of nonsynonymous variants based on PolyPhen2 [6], in association analysis of WES data [7–9]. On the other hand, genome-wide functional studies, such as the Encyclopedia of DNA Elements (ENCODE) project [10], have substantially advanced our knowledge about the functional DNA elements, especially noncoding regions, of the human genome. In contrast to the current practice of using the ENCODE information to annotate GWAS findings and prioritize functional variants to follow up [11], we propose to incorporate the ENCODE information in the discovery stage of association analysis of WGS data to boost the statistical power. Specifically, we use the RegulomeDB [12] scores as weights in existing RV association tests. RegulomeDB is a database that integrates a large collection of regulatory information of the human genomes, including multiple data sets such as ENCODE, expression quantitative trait locus (eQTL), computational predictions, manual annotation, and other sources, to identify functional variants and putative regulatory potential. The existing RV association tests we considered were the T5 burden test and SKAT, as a representative of unidirectional and omnidirectional tests, respectively. Although unidirectional tests assume that all the variants in a test unit, for example, a gene, influence the trait in the same direction, either increasing or decreasing, omnidirectional tests allow the presence of variants in both directions in a test unit [2]. We demonstrated the proposed framework using the WGS data and blood pressure phenotype from the San Antonio Family Studies (SAFS) provided by the Genetic Analysis Workshop (GAW) 19.

## Methods

### Genotype and phenotype data

We used the WGS data provided by GAW19 which included more than 8.3 million variants from odd-numbered chromosomes and 959 related individuals. The longitudinal phenotype data set had 1389 individuals including all samples with WGS data. Because there were many missing observations in the baseline measurement, we selected those subjects with at least 1 blood pressure measure among 5 visits and were able to obtain 789 related individuals. We used the earliest measurement among all completed visits for each person. We analyzed the systolic blood pressure (SBP) as a quantitative phenotype.

### Functional annotation of variants

We employed a sliding window approach to group RVs with a window length of 4 kb and a step size of 2 kb, as in Morrison et al. [5], resulting in 658,631 windows in total. The median number of variants in a window was 19. RegulomeDB provides a scoring system that categorizes variants by the confidence that a variant lies in a functional location and likely results in a functional consequence. There are 6 categories. Variants in category 1, which are supported by evidence from eQTL, transcription factor (TF) binding, matched TF motif, matched DNase footprint, and DNase peak, are considered to be most likely to affect binding and linked to expression of a gene target. Among the 8.3 million variants annotated in RegulomeDB, 0.26, 2.71, 2.12, 7.56, 30.76, and 56.59 % variants were assigned to category 1, 2, 3, 4, 5, and 6, respectively. If a nonsynonymous variant was not annotated by RegulomeDB, we used its PolyPhen2 functional prediction to assign it to a category, that is, “probably damaging” to category 1, “possibly damaging” to category 3, and “benign” to category 5. If a variant was not found in either RegulomeDB or PolyPhen2, we assigned it to category 6.

### Statistical methods

We used T5 and SKAT as the benchmark association tests of RVs with MAFs of less than 5 %. Given a quantitative trait $\boldsymbol{Y}=\left({Y}_1,,,\dots, {Y}_n\right)$ for *n* subjects, SKAT assumes a linear mixed effects model ${Y}_i={\gamma}_0+{\boldsymbol{Z}}_{\boldsymbol{i}}\boldsymbol{\gamma} +{\boldsymbol{G}}_{\boldsymbol{i}}\boldsymbol{\beta} +{\varepsilon}_i$, where ${\gamma}_0$ is an intercept; ${\boldsymbol{Z}}_{\boldsymbol{i}}$ is the *i-*th row vector of the covariate matrix; $\boldsymbol{\gamma} =\left({\gamma}_1,,,\cdots, {\gamma}_p\right)^{\prime }$ is a vector of fixed-effect coefficients; ${\boldsymbol{G}}_{\boldsymbol{i}}$ is the *i*-th row vector of the genotype matrix ***G*** coded as variant allele counts; $\boldsymbol{\beta} =\left({\beta}_1,,,\cdots, {\beta}_m\right)^{\prime }$ is a vector of random effects for RVs; and $\boldsymbol{\varepsilon} =\left({\varepsilon}_1,,,\dots, {\varepsilon}_n\right)\boldsymbol{^{\prime }}$ is a vector of random errors. Moreover, $\boldsymbol{\beta}$ follows an arbitrary distribution with $E\left[{\beta}_j\right]=0$ and $Var\left[{\beta}_j\right]={\omega_j}^2\tau$, and ${\omega}_j= Beta\left({MAF}_j,1,25\right)$ is a prespecified weight for variant $j$ ($j=1,..,m$). Thus the null hypothesis of no association between the phenotype and the $m$ RVs is reduced to ${H}_0:\;\tau =0$. As in Wu et al. [3], the SKAT test statistic under a linear kernel is ${T}_{SKAT}={\sum}_{j=1}^m{\omega}_j^2{\left({\sum}_{i=1}^n\left({Y}_i-{\widehat{\mu}}_i\right){G}_{ij}\right)}^2$, where ${\widehat{\mu}}_i$ is the predicted mean of ${Y}_i$ under ${H}_0.$ In addition to the default $Beta\left(1,25\right)$ weights assuming that rarer variants tended to have larger effect sizes, we used two other versions of weights: equal weights with all ${\omega}_j=1$ (called “uwSKAT”) and weights determined by RegulomeDB (called “regSKAT”) with ${\omega}_j^2=f\left({s}_j\right)$, where ${s}_j$ is variant $j$’s discrete functional category assigned by RegulomeDB. The function *f* transforms the RegulomeDB functional categories $\left(1,2,\cdots, 6\right)$ to numerical weights as detailed in the section “Transformation of functional categories” below. The T5 tests for the association between the phenotype and the mutation burden collapsed over the $m$ RVs defined as ${\sum}_{j=1}^m{\omega}_j{G}_{ij}$ in the linear regression framework, where ${\omega}_j=1$ if ${MAF}_j<0.05$ and ${\omega}_j=0$ otherwise [2]. We also applied 2 modified forms of the T5 test: the Madsen and Browning (MB) [2] weighting with ${\omega}_j=1/\Big({MAF}_j\left(1-{MAF}_j,\Big)\right)$ and the RegulomeDB weighting with ${\omega}_j=f\left({s}_j\right)$, called “regT5.”

We included age at visit, sex, smoking status, and blood pressure medication use as covariates in all the association analyses. In addition, because we analyzed the family-based samples in GAW19, we applied the above described tests, including SKAT, uwSKAT, regSKAT, T5, MB, and regT5, in the family-based SKAT and T5 frameworks [13]. As implemented in the R package “seqMeta,” family relatedness among individuals was properly taken into account by introducing a subject-specific random effect ${\delta}_i$, whose covariance matrix was proportional to twice the kinship matrix obtained from the pedigree information [13]. Using the conservative Bonferroni procedure for 658,631 sliding windows, we controlled the family-wise error rate (FWER) at 0.05 with a significance level $\upalpha$ = 0.05/658631 = 7.59e-08, which corresponds to $7.12$ on the $-{ \log}_{10}$ scale.

### Transformation of functional categories

As mentioned above, in regSKAT and regT5 we transformed the RegulomeDB discrete functional categories $\left(1,\;2,\dots,\;6\right)$ to numerical weights. We employed a quadratic function of the reverse order of categories $\mathrm{f}\left(\mathrm{s}\right)={\mathrm{s}}^2$, where s is the reverse order of a category; that is, s equals $6,\;5,\dots,\;1$ for category 1, 2,…, 6, respectively. We chose the quadratic transformation because it puts much less weight on low-confident functional categories, for example, 5 and 6, in which the variants are more likely to be neutral/nonfunctional. Of note, as majority of the sliding windows only included variants in categories 5 and 6, this weighting scheme was moderately informative for those windows, largely letting the observed genotype and phenotype data determine the association strength.

## Results

**Fig. 1:**
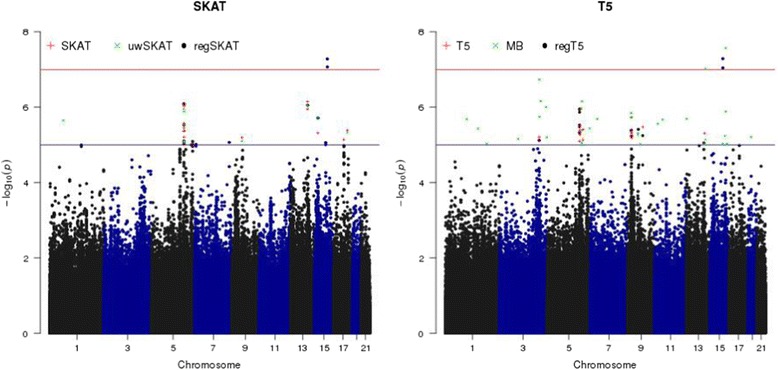
The Manhattan plots of $-{ \log}_{10}\left(\mathrm{p}\right)$ of regSKAT and regT5 for the odd-numbered chromosomes from 1 to 21. $-{ \log}_{10}\left(\mathrm{p}\right)$ of SKAT, uwSKAT, T5, or MB was also plotted if it was greater than 5. The red line corresponds to genome-wide significance threshold, while the blue line corresponds to a *p* value of 1e-05

**Fig. 2:**
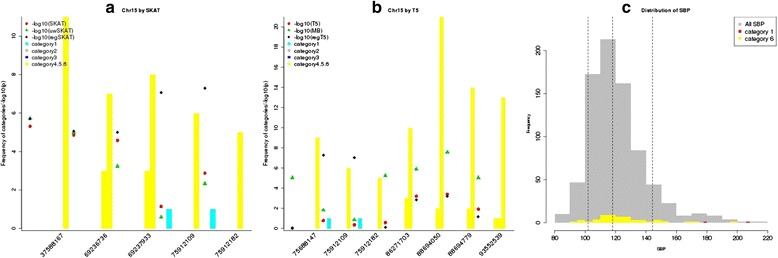
Functional annotation of the top windows on chromosome 15 and phenotype histogram for the genome-wide significant window. Panels **a** and **b** Bar plots showing the frequency of functional categories in the sliding windows that had at least 1 test with *p* value of <10^−5^ within each of the SKAT and T5 frameworks. X-axis corresponds to the center variant position in a sliding window. Dots in each window show $-{ \log}_{10}\left(\mathrm{p}\right)$. Panel **c** Histogram showing the distribution of SBP of 789 individuals. Carriers of the variants in the genome-wide significant window centering at variant chr15:75912109 were highlighted. Dotted lines indicate the 10th, 50th, and 90th percentiles of the observed SBP

As shown in the Manhattan plots (Fig. 1), regSKAT and regT5 identified some sliding windows on chromosome 15 with *p* values lower than the genome-wide significance threshold, while the *p* values for these windows by other tests that did not incorporate the ENCODE/PolyPhen2 information were far less significant. On the other hand, the MB T5 burden test also identified some genome-wide significant windows on chromosomes 13 and 15. We took a closer look at the significant sliding windows identified by regSKAT and regT5 on chromosome 15. Figure 2 shows the distribution of the functional categories in sliding windows with at least 1 *p* value less than ${10}^{-5}$ among the 3 methods in each of the SKAT and T5 frameworks. In particular, we observed that the genome-wide significant sliding windows centering at chr15:75912109 and chr15:75912182 included some variants in category 1, suggesting that the external biological information might have helped boost the signals. We further looked into the sliding window centering at chr15:75912109, which included a doubleton variant chr15:75913349 in category 1 and a few other variants in category 6. All the variants in this window were annotated to gene *SNUPN*, standing for snurportin 1, which has not been reported to be associated with blood pressure. It turned out that exonic variant chr15:75913349 was not annotated in RegulomeDB, but was annotated as a probably damaging nonsynonymous variant by PolyPhen2 with a confidence score of 99.2 %, resulting in category 1 in our weighting scheme. This variant was also predicted to be highly deleterious by several other functional prediction algorithms including sorting tolerant from intolerant (SIFT), likelihood ratio test (LRT), and MutationTaster [14]. As shown in the histogram in Fig. 2c, 2 individuals who were half-siblings and carriers of nonsynonymous variant chr15:75913349 had SBP of 179 and 208, respectively, with the latter close to the maximum observed SBP. Although the effect sizes of the rest variants in this window were not as large as that of chr15:75913349, the carriers tended to have higher SBP. As all the variants increased the SBP, that is, in the same direction of effect size, regT5 was able to identify this sliding window as well. We noted that the MB T5 burden test also identified 2 significant sliding windows centering at chr13:96267813 (near gene *DZIP1*) and chr15:88694779 (near gene *NTRK3*), respectively, although neither gene was reported to be associated with blood pressure before. As all variants were assigned to low functional categories, that is, 5 and 6, in these windows, T5 and regT5 gave nonsignificant *p* values of similar magnitudes (see Fig. 2b). A closer look revealed that these windows contained some rarer variants, for example, doubletons, whose carriers tended to have higher SBP, favoring the assumption of the MB weighting scheme. This suggested that in the absence of informative external biological knowledge, the MAF might provide useful information.

To investigate if the proposed weighting scheme might increase the false-positive rate when there was no association, we used the simulated phenotypes provided by GAW19 to evaluate the type I error rate. We randomly selected 50 sliding windows on chromosome 15 that did not include any causal variants in the GAW19 simulation model. At the significance level α = 0.05, the empirical type I error rates averaged over 50 sliding windows and 200 simulated phenotype sets were 0.0551, 0.0506, 0.0513, 0.0506, 0.0535, and 0.0467 for SKAT, uwSKAT, regSKAT, T5, MB, and regT5, respectively, suggesting that incorporating external biological information into existing RV association tests did not inflate the type I error.

## Discussion

We have proposed a general framework to exploit external biological information in the analysis of WGS data. We identified a genome-wide significant locus on chromosome 15 harboring a rare nonsynonymous variant, while other methods without leveraging biological information did not identify it. This significant locus warrants following up and replication in future independent studies.

The proposed general framework can be used to incorporate other genome-wide functional annotations and conservation scores, such as CADD [15] and GERP++ [16]. Given that these functional annotation systems are likely to be incomplete because of limited biological knowledge, they may provide complementary information and it would be of interest to integrate multiple functional scores simultaneously. In addition, alternative weighting schemes other than the one proposed here would be worth investigating regarding the power and Type I error rate.

In this study, we used the Bonferroni procedure to correct for multiple testing. Considering that the neighboring sliding windows overlapped with each other, the Bonferroni correction was very conservative, leading to reduced statistical power. Further research is warranted to estimate the effective number of tests in the sliding window framework. Finally, we adjusted the treatment effect on the SBP by simply including the medication use as a binary covariate in the regression framework; alternative adjustment methods as studied in Tobin et al. [17] are worth investigating.

## Conclusions

In summary, we have proposed a general framework to incorporate the ENCODE and PolyPhen2 information into association tests of WGS data. We demonstrated the potential statistical power gains with the proposed method using the GAW19 WGS genotype and SBP phenotype data. Because it remains a challenge to analyze WGS data, it would be worth capitalizing on newly available biological knowledge in the proposed and alternative frameworks to maximize the power of genomic discovery.
